# Supplementary figures and images for: Live-attenuated pediatric parainfluenza vaccine expressing 6P-stabilized SARS-CoV-2 spike protein is protective against SARS-CoV-2 variants in hamsters
Source: PLoS Pathog. 2023 Jun 23;19(6):e1011057. doi: 10.1371/journal.ppat.1011057 (PMC10325082; doi:10.1371/journal.ppat.1011057)

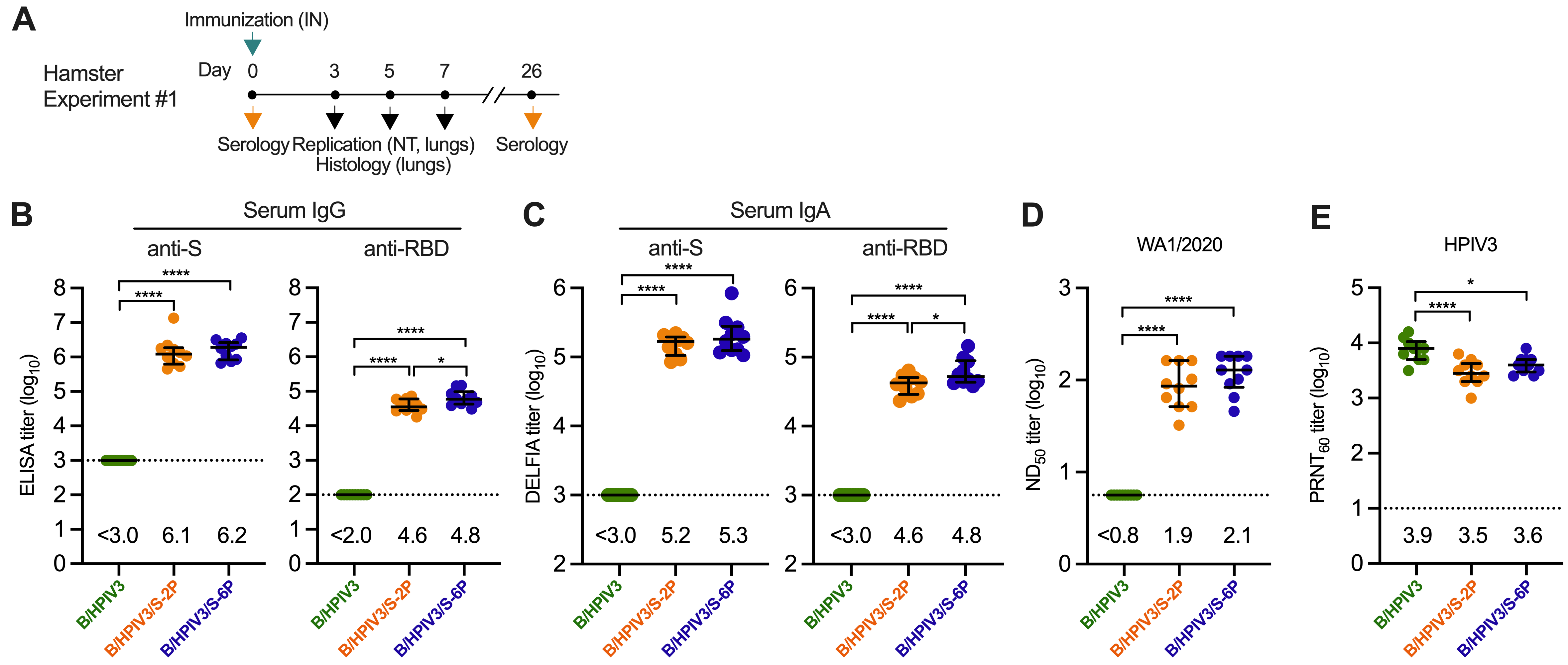

Supplement: S1 Fig — (A) Schematic overview of Experiment #1. Hamsters were immunized intranasally with 5.0 log10 PFU of B/HPIV3, B/HPIV3/S-2P, or B/HPIV3/S-6P. Vaccine virus titers detected in respiratory tissues on days 3, 5, and 7 after immunization are shown in Fig 2. Sera from n = 10 animals per group were collected on day 26 after immunization. (B) IgG ELISA titers to a secreted form of the S-2P protein or to a fragment of the S protein (aa 328–531) containing SARS-CoV-2 receptor-binding domain (RBD) and (C) IgA titers to the S-2P or RBD, determined by dissociation-enhanced lanthanide time-resolved fluorescence (DELFIA-TRF) assay. (D) The 50% SARS-CoV-2 neutralizing doses (ND50) were determined on Vero E6 cells against the vaccine-matched strain WA1/2020. (E) Sera were also analyzed to determine the 60% plaque reduction neutralization titers (PRNT60) to HPIV3. Each hamster is represented by a symbol and medians and interquartile ranges are shown. GMTs are indicated above the x axis and the limit of detection is indicated by a dashed line. * = P<0.05; **** = P<0.0001 (One-way ANOVA with Tukey’s multiple comparisons). (TIF) [file ppat.1011057.s002.tif]

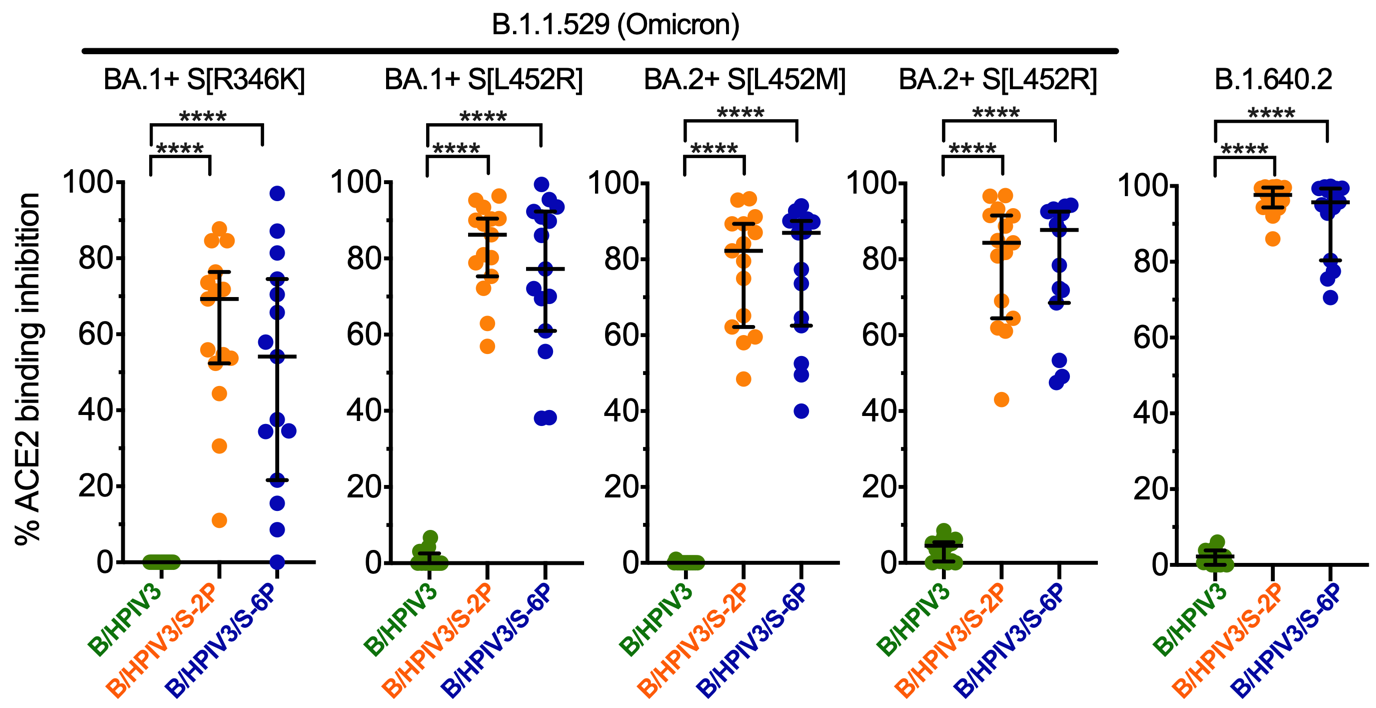

Supplement: S2 Fig — An ACE2 binding inhibition assay was used as an alternative for a BSL3 live-virus neutralization assay. Heat-inactivated hamster sera were diluted 1:20 and added to duplicate wells of 96-well plates spot-coated with the indicated S proteins. The percent binding inhibition of Sulfo-tag labelled ACE2 to S proteins of the indicated variants by serum antibodies from immunized hamsters was determined by electrochemiluminescence. Each hamster is represented by a symbol, and medians and interquartile ranges are shown. **** = P<0.0001 (One-way ANOVA with Tukey’s multiple comparisons). (TIF) [file ppat.1011057.s003.tif]

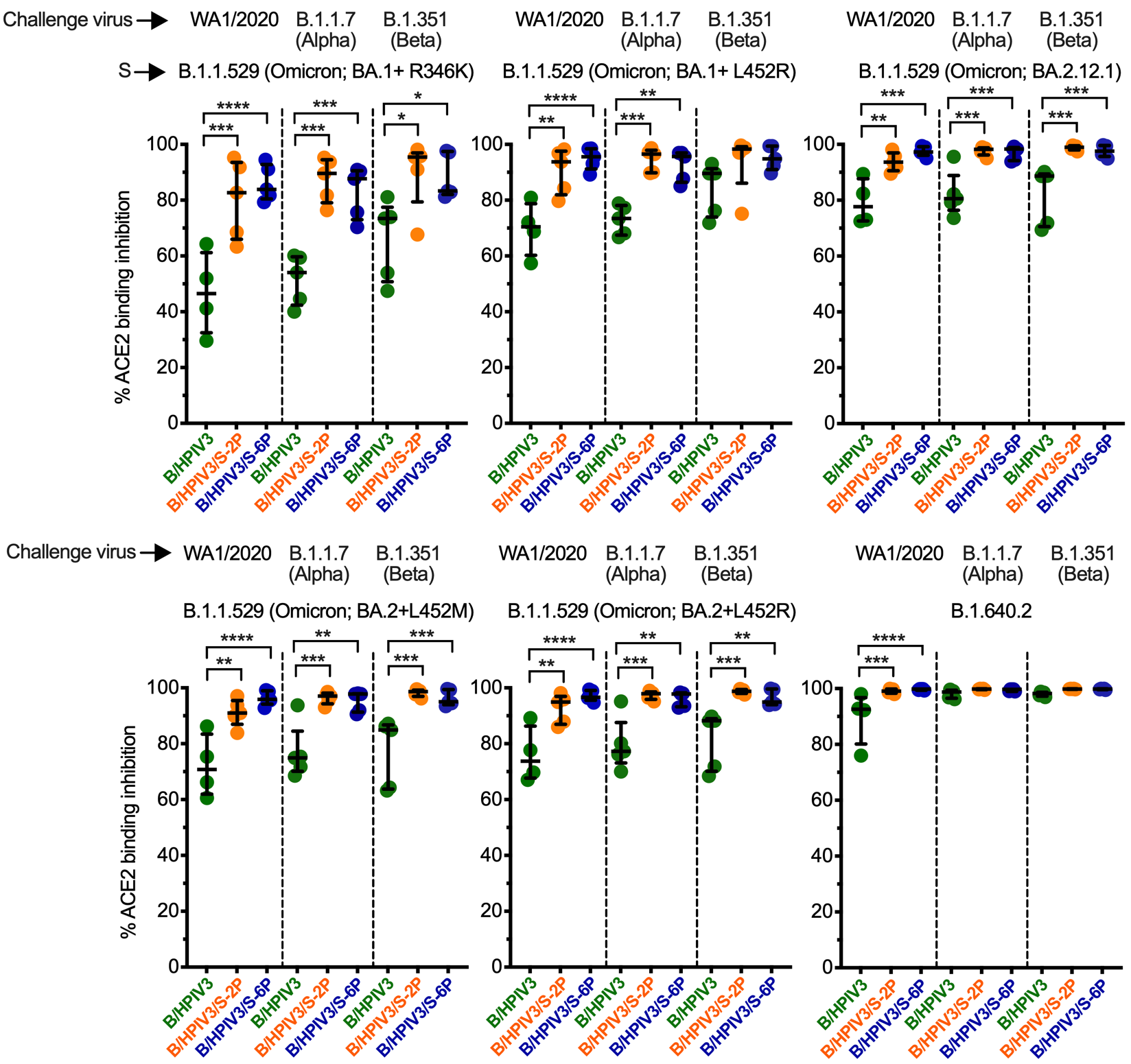

Supplement: S3 Fig — An ACE2 binding inhibition assay was used as an alternative for a BSL3 live-virus neutralization assay. Heat-inactivated hamster sera were diluted 1:20 and added to duplicate wells of 96-well plates spot-coated with the indicated S proteins. The percent binding inhibition of Sulfo-tag labelled ACE2 to S proteins of the indicated variants by serum antibodies from immunized hamsters was determined by electrochemiluminescence. Each hamster is represented by a symbol, and medians and interquartile ranges are shown. * = P<0.05; ** = P<0.01; *** = P<0.001; **** = P<0.0001 (One-way ANOVA with Sidak’s multiple comparisons). (TIF) [file ppat.1011057.s004.tif]
